# Penetration and Tensile Strength of Various Impression Materials of Vinylsiloxanether, Polyether, and Polyvinylsiloxane Impression Materials

**DOI:** 10.1055/s-0041-1735793

**Published:** 2021-12-01

**Authors:** Pongsakorn Apinsathanon, Bishwa Prakash Bhattarai, Suphachai Suphangul, Natthamet Wongsirichat, Napapa Aimjirakul

**Affiliations:** 1 Department of Conservative Dentistry and Prosthodontics, Faculty of Dentistry, Srinakharinwirot University, Bangkok, Thailand; 2 Walailak University International College of Dentistry, Phaya Thai, Bangkok, Thailand; 3 Department of Advanced General Dentistry, Faculty of Dentistry, Mahidol University, Bangkok, Thailand

**Keywords:** penetration ability, tensile strength, elastomeric impression materials, gingival sulcus width, moist environment, various impression materials

## Abstract

**Objectives** The aim of this study was to evaluate and compare penetration ability and tensile strength among vinylsiloxanether (VSE), polyether (PE), and polyvinylsiloxane (PVS) elastomeric dental impression materials.

**Materials and Methods** The models were constructed for penetration ability test by simulated gingival sulcus width and moist environment. The 0.05, 0.1, and 0.2 mm of simulated gingival sulcus widths were used. Each simulated gingival sulcus width was impressed 10 repeats per one elastomeric impression material. All extension of elastomeric dental impression materials was scaled by Measuring Microscope (MM-11; Nikon, Tokyo, Japan). On the issue of the tensile strength study, the models were constructed following type 1 of the ISO 37:2017 specifications and/or type C of ASTM.D412 specifications. The two-way analysis of variance (ANOVA) and Tukey's honest significant difference test were performed in the penetration ability test. The one-way ANOVA and Dunnett's T3 test were performed in the tensile strength test. The significance level was set at 0.05.

**Results** PE showed the best extension into all widths of simulated sulcus followed by VSE and PVS, respectively. PVS was significantly higher in tensile strength than VSE and PE, while VSE was significantly higher than PE.

**Conclusion** Penetration ability of elastomeric dental impression materials was depended on gingival sulcus width. The wider the sulcular width, the better the penetration ability of elastomeric dental impression materials. PE presented the best penetration ability, while the novel PVS showed highest tensile strength.

## Introduction

The dental impression material is the key to replicate all details which are needed for fabricating a dental indirect restoration. Good quality of dental impression material brings good imitating of the area in which restoration is needed. Impression material in dentistry generally requires accuracy, elastic recovery, dimensional stability, hydrophilic, flow or rheological properties, flexibility, and deformation. 1 In addition to the general properties of dental impression material, the ideal properties included long shelf life, patient comfort, and economics. 2

Polyether (PE), Impregum (ESPE GmbH, Germany), was the first elastomeric impression material developed for dentistry. 3 Some said it was introduced in the late 1960s, 4 while some said it was introduced in the late 1970s. In the beginning, it has only single regular viscosity. 3 The material is hydrophilic, so it can be used in humid surroundings. In addition, good wettability offers dental gypsum flow easier on the impression resulting in good detail of dental cast. 5 PE has very good elastic properties but very rigid when the material is completely set. Hence, it may be difficult to be used in a patient with many embrasures and commonly causing fracture of die gypsum. However, novel PE impression materials are manufactured to be more flexible than the original one when completely set. Comparing to the original PE, the new PE is easier to be removed from patient's mouth and more secure to remove die gypsum from the completely set impression. 6 As a result of the characteristic of the material absorbing water, the impression should not immerse in water for a period of time as it could lead to swelling of the impression. 7 On the other hand, Guiraldo et al 8 founded that PE had good dimensional stability even immersed the material for 15 minutes under disinfectant. PE, by nature, has no by-product of setting reactions resulting in good dimensional stability. 3

In the 1970s, polyvinylsiloxane (PVS) was introduced to the dental world and become popular because of best dimensional stability due to no by-product while setting 3 4 and more elastic after setting than PE. Kettenbach Company has launched the newest impression material called vinylsiloxanether (VSE) “Identium” and claims that this material combined the advantage of PE and additional type silicone impression material in 2009. 9 Even manufacturers postulate that PE and VSE have a hydrophilic property and good at challenging environments, the previous study showed that all elastomeric impression materials need a dry environment for a good performance. 10 However, the study of the properties of this material still scant. 11

This leads to the reason for the study which is to compare the penetration ability and tensile strength of different elastomeric dental impression materials including VSE, PE, and PVS *in vitro* for that the knowledge could be applied for clinical uses. The null hypothesis was the penetration ability and tensile strength among elastomeric impression materials which were not different.

## Materials and Methods

The materials used in this experiment were VSE, PE, and PVS. All kinds of elastomeric impression material used in heavy body and light body are shown in Table 1 .

**Table 1:** Elastomeric dental impression materials tested

Type of impression materials	Brand name	Consistency	Lot number
Vinylsiloxanether	Identium, Kettenbach	Heavy body	14724
		Light body	200281
Polyether	Impregum, 3M ESPE	Heavy body	5023235
		Light body	7478882
Polyvinylsiloxane	Imprint4, 3M ESPE	Heavy body	7288387
		Light body	7369858

### Model Construction

#### Penetration Ability Test

**Fig. 1:**
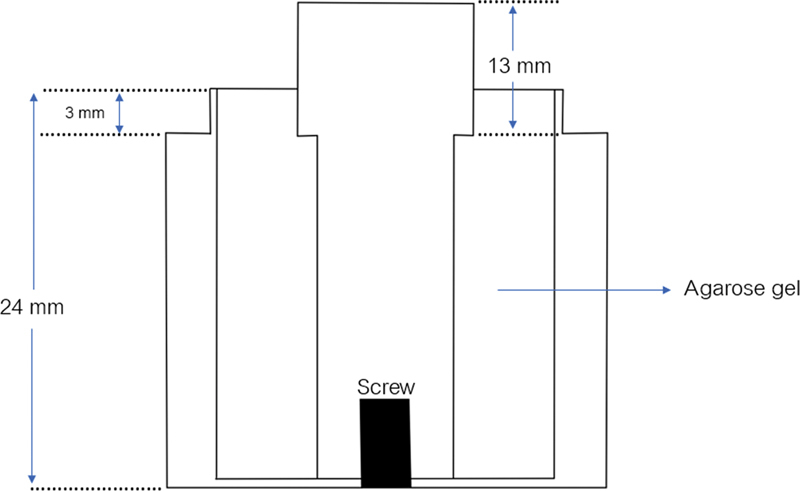
Model to simulate gingival sulcus with cylindrical-shaped stainless steel.

**Fig. 2:**
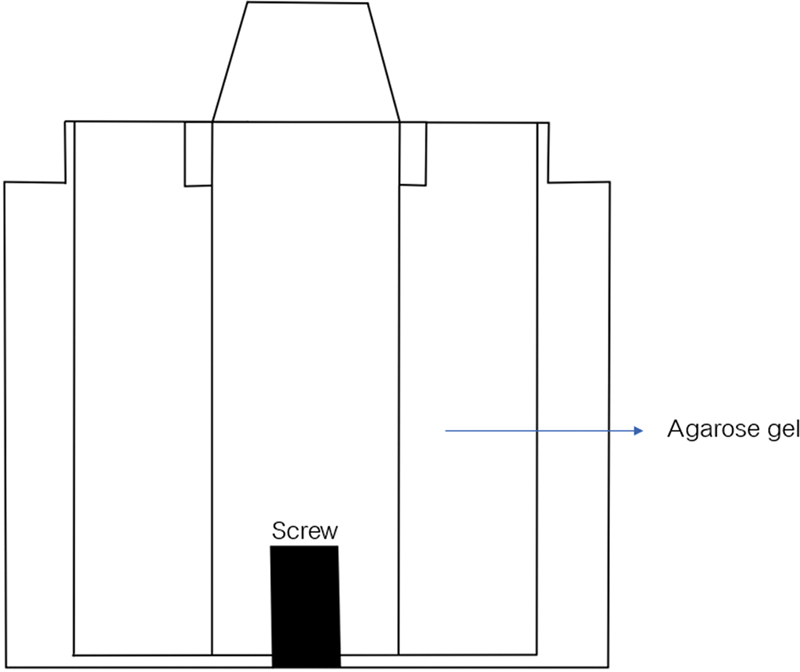
The model of simulating gingival sulcus. One per cent agarose gel represents gingival tissue. The cylindrical-shaped stainless steel representing a tooth.

The cylindrical-shaped stainless steel of size 10.4, 10.2, and 10.1 mm in diameter was screwed in the plastic block to simulate sulcus. One percent agarose gel was poured into the space between cylindrical-shaped stainless steel and plastic block in an incubator which controls 27 ± 2°C and 100% relative humidity ( Fig. 1 ). The screw at the bottom of the plastic block was loosen and then removed the cylindrical-shaped stainless steel. The result is simulating the gingival tissue of 1% agarose gel. After removing the cylindrical-shaped stainless steel, the new cylindrical-shaped stainless steel with chamfer finishing line 0.5 mm and 3 degrees convergence was put into the plastic block to represent the prepared tooth. The sulcus depth of 3 mm and the sulcus width of 0.2, 0.1, and 0.05 mm were made ( Fig. 2 ). The model was used immediately for penetration ability tests after construction.

#### Tensile Strength Test

**Fig. 3:**
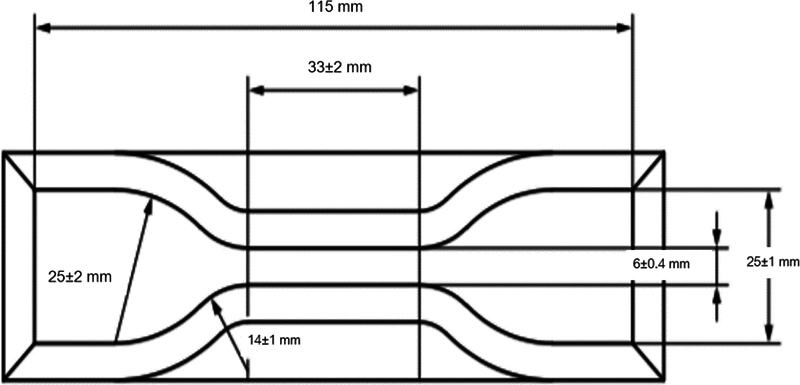
The dumbbell-shaped according to the type 1 of the ISO 37:2014 specifications and/or type C of ASTM.D412 specifications.

The mold of the dumbbell-shaped specimen was milled according to the type 1 of the ISO 37:2017 specifications 12 and/or type C of ASTM.D412 specifications, 13 as shown in Fig. 3 .

### Method

#### Penetration Ability Test

**Fig. 4:**
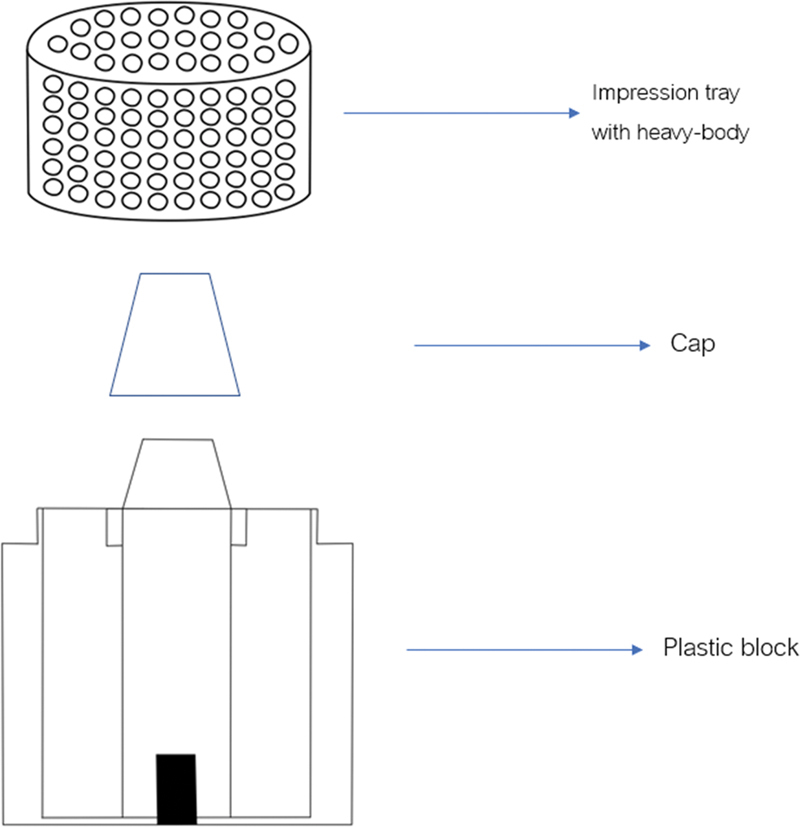
The impression tray, cap, and plastic block.

**Fig. 5:**
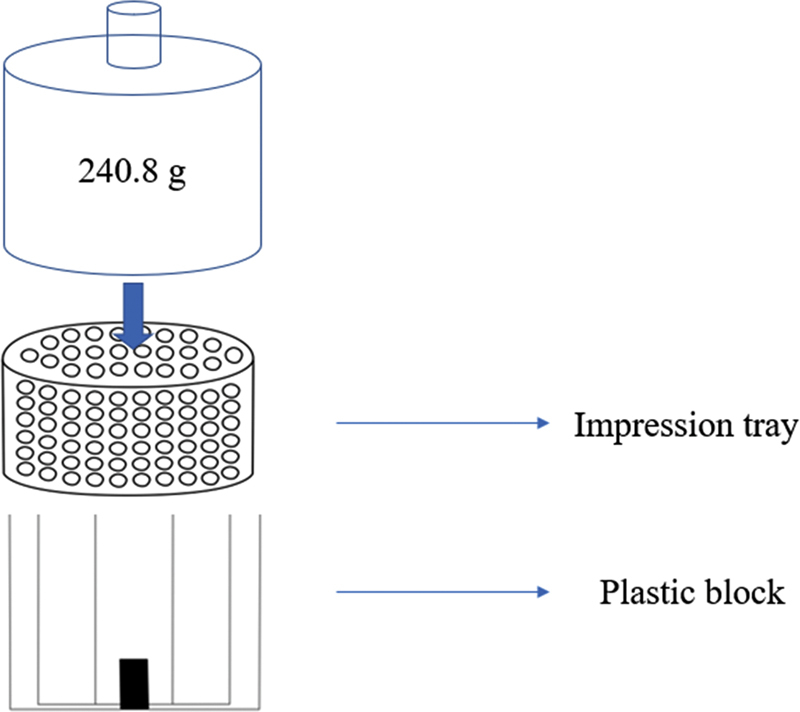
The 240.8 g of pendulum was placed on impression tray and plastic block.

**Fig. 6:**
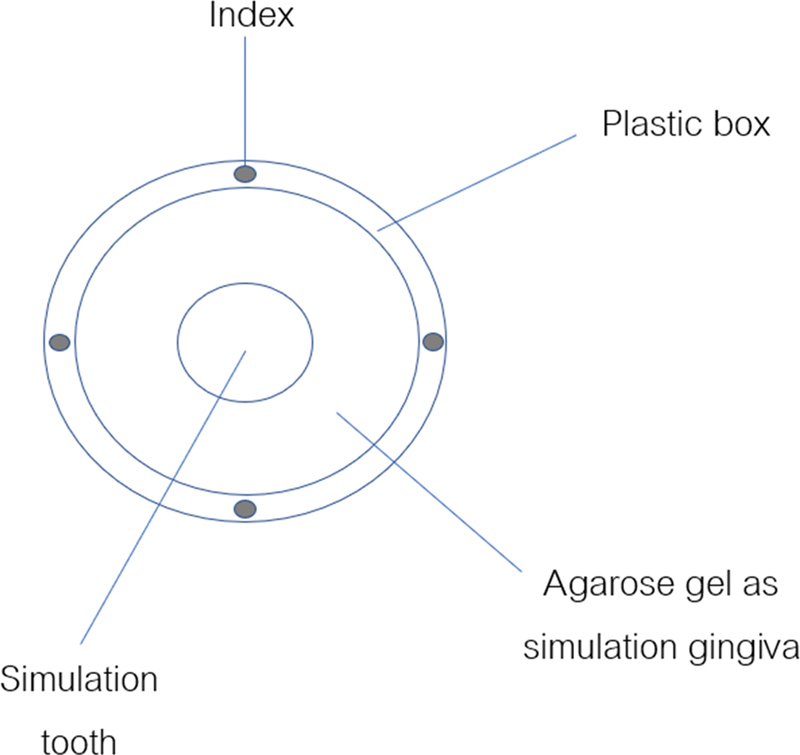
Four-point index on the plastic block.

A double-mix, double-impression technique was used with all impression material with heavy-body and light-body impression materials. All impression samples were done using the syringe-tray technique (Pentamix 3, 3M ESPE). Each of the heavy-body impression material was injected into an impression tray by the tip of the syringe always immersed in the impression materials to prevent void formation. At the start of a new cartridge and before every new mixing process, first, extrude a new quantity of paste without using a mixing tip until both components emerge evenly as described by the manufacturer. Excess impression material over the edge of the impression tray was wiped off by spatula to make impression material fit the margin of the impression tray. The cylindrical-shaped stainless steel was covered by a cap made from plastic by milling procedure for relieving space 1 mm, as shown in Fig. 4 . The impression tray was immediately seated on the plastic block by pushing force of an operator. The heavy-body impression materials were waited until fully set longer than recommended by the manufacturer. The impression tray was taken off from the plastic block and remove plastic cap from heavy body. The light-body impression material was immediately injected on fully set heavy-body impression material by the tip of the syringe always immersed in the impression materials to prevent void formation. The impression tray was gently seated on the plastic block. Then, a pendulum with 240.8 g of weight was placed on the impression tray, as shown in Fig. 5 . The light-body impression material was waited until fully set longer than recommended by the manufacturer. The pendulum was removed. The impression tray was taken off from the plastic block. The sample was stored at room temperature for 30 minutes before measuring an extension of the impression. The extension of the light-body impression material that represents penetration ability was measured by Measuring Microscope (MM-11; Nikon, Tokyo, Japan) using four reference points at the margin of the plastic block, as shown in Fig. 6 , which was replicated by the impression material. All these steps were performed at room temperature, which is 27 ± 2°C, according to the standard laboratory temperature of ISO 23529. 14 The penetration ability of each sample was measured in millimeter four times according to four reference points, as shown in Fig. 6 . Four values for each sample were added together, then divided by four. This value stands for the penetration ability of the sample.

#### Tensile Strength Test

Two pieces of a screw were tightened at the same site (top or bottom) for forming the base of the mold. Each of the light-body impression materials was injected into the space of the mold by the tip of the syringe always immersed in the impression materials to prevent void formation. The remaining two screws were tightened to cover the light-body impression material. The light-body impression material was waited until fully set longer than the manufacturer's recommendations. All four screws were then loosened. The light-body impression material was removed from the mold. The creeping excess was removed from a specimen. The sample was stored at room temperature for 30 minutes before testing the tensile strength of the impression material. The ultimate tensile strength of the light-body impression materials was tested by a Universal Testing Machine (EZ Test; Shimadzu Corporation, Kyoto, Japan). All these steps were performed at room temperature, which is 27 ± 2°C, according to the standard laboratory temperature of ISO 23529. 14

### Statistical Analysis

For penetration ability test, two-way analysis of variance (ANOVA) was performed on the data set, with P set at .05. Where significant differences in the groups were found, individual means were compared with the multiple comparison test. For tensile strength test, one-way ANOVA and multiple comparison test were performed with the *p* -value at 0.05. The normality of deviations was evaluated using the Shapiro–Wilk's test and the homogeneity of variance was assessed by using the Levene's test. Statistical Package of Social Sciences version 18 (IBM SPSS Statistics for Windows, Version 18, New York, United States) was used with the *p* -value at 0.05.

## Results

For the penetration ability, Table 2 showed the mean values and standard deviations of penetration of light body of each impression materials. Considering the same material, various simulated sulcular width, VSE, PE, and PVS impression materials in this study showed the longest distance of penetration at 0.2 mm of simulated sulcular width followed by 0.1 and 0.05 mm, respectively. Regarding the simulated sulcular width at 0.05 mm, VSE had no significant difference in penetration ability with PE and PVS, while PE had significant difference in penetration ability with PVS. Respecting the 0.1 mm of simulated sulcular width, VSE had no significant difference in penetration ability with PE, whereas both VSE and PE had significant difference in penetration ability with PVS. At the simulated sulcular width 0.2 mm, VSE, PE, and PVS had significant difference in penetration ability with one another. PE indicated greater penetration ability followed by VSE and PVS, respectively, in any simulated sulcular width, in this study, together with 0.2 mm of simulated sulcular width had shown the best extension of all impression. For tensile strength, Table 3 reveals the mean values and standard deviations of tensile strength of each impression materials. PVS had the best tensile strength followed by VSE and PE, respectively.

**Table 2:** Mean values and standard deviations of penetration of light body of each impression materials

Width of simulated sulcus (mm)	Identium Kettenbach		Impregum 3M ESPE		Imprint 4 3M ESPE	
	Mean	SD	Mean	SD	Mean	SD
0.05	0.185 ^A,a,b^	0.042	0.213 ^A,a^	0.036	0.166 ^A,b^	0.031
0.1	0.447 ^B,a^	0.040	0.520 ^B,a^	0.174	0.279 ^B,b^	0.040
0.2	0.846 ^C,a^	0.030	0.899 ^C,b^	0.024	0.785 ^C,c^	0.016

Abbreviation: SD, standard deviation.

Note: Groups with the same uppercase superscript letter indicated no significant differences between simulated sulcular widths in the same impression material at *p* < 0.05. Groups with the same lowercase superscript letter indicated no significant differences between impression materials in the same simulated sulcular widths at *p* < 0.05.

**Table 3:** Mean values and standard deviations of tensile strength of each impression materials

	Identium Kettenbach	Impregum 3M ESPE	Imprint 4 3M ESPE
Mean	31.0100	15.2127	47.3625
Standard deviation	1.7186	1.5742	8.0519

## Discussion

Different types of elastomeric impression materials consist of various components and structure results in distinct property of elastomeric impression materials. This study showed that the penetration ability of PE was highest, whereas PVS had lowest penetration ability under experimental condition. In term of tensile strength, PVS showed highest force, while PE showed lowest force in tensile strength test condition.

PE is moderately hydrophilic 1 dental impression material by nature. This lets PE work better than other elastomeric impression materials in the moist environment. Even though PE can work well in some wetting situation, clinician should prepare a dry environment to make acceptable impression. Correspond to this study, when simulated gingival act as moist situation, PE showed the greatest extension.

On the contrary, PVS demonstrated lowest penetration ability in this study. Naturally, PVS is hydrophobic. The novel PVS has more hydrophilic by improving wettability 15 ; however, the impression of PVS is only acceptable in dry condition. 16 These are some reasons that lead PVS presented bad penetration ability in this experiment.

The penetration ability in this study delineated only by double-mix, double-impression technique. Notwithstanding, the extension of dental impression material is not only upon the impression technique but many influencing factors are also involved. The ideal dental impression material should consist of good accuracy, good elastic recovery, good dimensional stability, good flowability, good flexibility, good workability, hydrophilicity, long shelf life, patient comfort, and reasonable in economic. 2

As mentioned earlier, generally, the hydrophilic property plays important role in humid condition. Hydrophobic, such as PVS, displays contact angle 90 degrees or more with water, while hydrophilic type of dental impression materials, such as PE, presents a lower contact angle. 1 This is one of the reasons that can explain why PE revealed the greatest elongation, while PVS illustrated the lowest in this study. The newly PVS impression material improved hydrophilic property by reducing contact angle with water. 17 Widely known, contact angle is measured by dropping a drop of water on a surface of material then scale the angle between a drop of water and material's surface. Therefore, the lower contact angle with water represents the better flowability of water on material's surface. With this knowledge, we can imply that the new PVS which has lower contact angle can be compatible with a dental stone; however, it does not flow properly in the narrow gingival sulcus because of its hydrophobic property. 15 Unlike PE, PE has hydrophilic property on its own resulting in better flow in damp areas. VSE seems to be average penetration ability among elastomeric impression materials used in this study. Rupp et al 18 and Van Krevelen and Te Nijenhuis 19 disclosed that chemical structures of PE and VSE have more hydrophilic than PVS. Because of interweaving of hydrophilic and hydrophobic chemical structures, VSE represented moderate penetration ability in this experiment. Even Kettenbach notify that do not use Identium impression materials for two-step putty-wash impression technique, but we used this impression technique in this study and did not notice deformity, cohesive failure between light body and heavy body or another failure of the impression material. Baer 20 stated that Identium has been optimized for the one-step impression technique or monophase technique. These might result from this hybridization material allows a long working time (a desired characteristic of PEs) and a short setting time (a desired characteristic of PVSs); hence, it is not necessary to take an impression with two-step double-mix, double-impression technique.

Aside from dental impression material's properties, gingival sulcus width affects in quality of dental impression. Laufer et al 21 suggested that the minimum of sulcus width which can produce good impression was 0.2 mm. Despite Ramadan 22 measured sulcular width immediately after the removal of medical retraction cord at 0.3 to 0.4 mm, Laufer et al revealed that the gingival sulcus will recover from 0.37 to 0.24 mm of sulcular width after the removal of retraction cord (No. 1 Ultrapak retraction cord; Ultrapak, Salt Lake City, Utah, United States) in 20 seconds. 23 Moreover, the previous study 24 measured a gingival sulcus width immediately after removing retraction cord (No.00 knitted cord impregnated with 15.5% ferric sulfate; Ultradent products, United States) and displacement paste (Expasyl; Pierre Rolland, France) which were 0.21 ± 0.01 mm and 0.26 ± 0.02, respectively. We can imply that a suitable time to taking an impression is within 20 seconds after removing a retraction cord and a final retraction cord should be bigger than No.00. We had applied this knowledge with our study by various simulated gingival sulcus into 0.2, 0.1, and 0.05 mm for evaluating penetration ability of the dental impression materials, as seen in Table 2 . We noticed that different simulated gingival sulcus effect on penetration ability of each dental impression materials in the study. At 0.05 mm of simulated gingival sulcus width, PE dental impression material which was Impregum in this study had shown the greatest extension followed by VSE which was Identium (Kettenbach), and PVS which was Imprint 4, respectively. The extension had statistically significant difference between PE and PVS, but no statistically significant difference between VSE and PE. Like the correlation between VSE and PE in this study, VSE had no statistically significant difference in penetration ability with PE at 0.05 mm simulated gingival sulcus width compared with the results from the study by Aimjirakul et al, 25 which were not significantly different among the penetration ability of elastomeric impression materials in the study. The different could result from we studied on developed elastomeric impression materials and developed model. However, we could agree that no elastomeric impression materials able to capture the details at 0.05 mm of sulcular width. Simulated gingival sulcular width at 0.1 mm, PE still showed the best extension into simulated gingival sulcus. No statistically significant difference between VSE and PE was observed, but both had statistically significant difference with PVS. Considering at 0.2 mm of simulated gingival sulcus width, PE still showed the best result followed by VSE and PVS, respectively, like the results of 0.05 and 0.1 mm of simulated gingival sulcus width, but all were statistically significantly different. Moreover, Chauhan et al 26 founded that PE had the best accuracy among all elastomeric impression materials. Together with the previous study, 27 the research showed that PE had more accuracy for reproduction a detail of a surface of impression compared with the VSE and PVS impression materials. Although the results show that PE works well in terms of penetration ability at all simulated sulcus widths, Laufer et al 21 founded that significantly distortion occurred with all elastomeric impression materials tested with sulcular widths less than 0.2 mm. The study, also, suggested that none of elastomeric impression materials was suitable for use in sulcus width 0.05 mm because of the high prevalence of tears.

On the contrary, we have been believed that PE can be stiffer when fully set than other dental impression materials, 1 but in this study, we found that the novel PVS which was Imprint 4 had highest tensile strength among all elastomeric impression materials studied. We can imply that the new PVS has the best tear strength among elastomeric impression materials studied.

There is no standard protocol from any organization such as the American Dental Association on how to measure the penetration ability for nonaqueous, elastomeric dental impression materials. 28 All models which study penetration ability of elastomeric dental impression materials were invented by a researcher, including the shark fin test. This study had developed a model that mimic an oral cavity conditions, unlike the shark fin test that represents only on the flowability of the impression materials. 29 The stainless steel which has 0.5 mm chamfer margin with slightly taper was an ideal tooth preparation for dental crown restoration and the agarose was a good representation of gingiva in moist condition. The patent number 75862 at the Department of Intellectual Property, Bangkok, Thailand was registered in 2020 for the model and was used in this study. 30

For clinical application, a clinician should retract gingival sulcus at least 0.2 mm for acceptable impression. For the crown with undercut, a clinician should use PVS instead of PE which suggested to use in normal crown. The study model which imitates more oral condition such as a simulation of gingival fluid pressure may need for the further study to get closely clinical situation.

## Conclusion

Within the limitations of this laboratory study, we can conclude that the wider the gingival sulcus width, the greater the penetration ability of the elastomeric dental impression materials. The sulcular width suitable for all elastomeric impression material is 0.2 mm or wider because of the prevalence of penetration. While PE shows the best penetration ability, the novel polyvinylsiloxane has the highest tear resistance.

### Authors' Contributions

**Table:** 

Conceptualization	N.A., P.A.
Methodology	N.A., P.A.
Validation	N.A.
Formal analysis	P.A.
Investigation	P.A.
Resources	N.A.
Data curation	P.A.
Writing—original draft preparation	P.A.
Writing—review and editing	B.P.B., S.S.
Visualization	P.A.
Supervision	N.A., N.W.
Project administration	N.A.
Final approval	N.W., N.A.
Agreement to accountable	N.A.
